# Efficient in-situ synthesis of heterocyclic derivatives from benzyl alcohols using pyrazinium chlorochromate-functionalized carbonitride as a novel catalyst

**DOI:** 10.1038/s41598-024-75036-6

**Published:** 2024-10-14

**Authors:** Hasan Soltani, Zeinab Tajik, Zahra Nasri, Peyman Hanifehnejad, Elaheh Hamidi, Zahra Aslbeigi, Hossein Ghafuri

**Affiliations:** Catalysts and Organic Synthesis Research Laboratory, Department of Chemistry, Iran University of Science and Technology, Tehran, 16846-13114 Iran

**Keywords:** Heterogeneous catalyst, CNs@PCC, 3,4-dihydropyrimidin-2-(1*H*)-one, 1,4-dihydropyridine, Chemistry, Materials science

## Abstract

The synthesis of efficient organic compounds from simple substrates is both noticeable and important. However, it can be challenging to achieve this target using suitable strategies. To address this issue, pyrazinium chlorochromate (PCC) was used to modify carbonitride nanosheets (CNs) and applied as a heterogeneous catalyst in the oxidation of benzyl alcohols. That can be regarded as innovation in ingenious synthesis of the PCC on the CNs. Then, it was identified by varied techniques such as EDS, FT-IR, XRD, STA and FE-SEM. This process resulted in in-situ synthesis of 1,4-dihydropyridine and 3,4-dihydropyrimidin-2-(1*H*) one derivatives with excellent yield.

**Supplementary Information:**

The online version contains supplementary material available at 10.1038/s41598-024-75036-6.

## Introduction

Aldehydes are beneficial organic compounds that can be widely utilized various applications such as tanning, preserving, and acting as germicide, fungicide, and insecticide for plants. on the other side, they are extensively used in the synthesis of polymeric materials^1^. Aldehydes can also be converted into more valuable materials and heterocyclic compounds like 1,4-dihydropyridine ,3,4-dihydropyrimidin-2-(*1 H*)-one, and dihydroquinazoline, which have high medicinal activity^2–4^.

Therefore, the selection of a suitable method for the production of desired aldehyde is still challenging and considerable. Oxidation of alcohols is one of various methods for the production of corresponding aldehydes using different reagents such as MnO_2_, PCC (Pyridinium chlorochromate), CrO_3_/H^+^, Swern (DMSO, (COCl_2_), Et_3_N), and so on. Hence, the Cr (VI) oxidants react selectively and do not oxidize alkynes and alkenes as a result can considered as appropriate oxidants for this purpose^5,6^. But the presence of H_2_O and the use of oxidants that are strong enough can be two causes of concern for converting aldehyde to carboxylic acid, accordingly, control of reaction condition and an excellent oxidant is noticeable for synthesis of aldehydes with high yield^7^. For this target, the PCC reagent can be an excellent selection because it is not strong enough to convert alcohol to carboxylic acid^8,9^.

Although, there are several problems in applying this reagent like hard separation and the use of toxic solvents by use of a great strategy can be excellently rectified. To modify the foregoing condition, if it is linked to a heterogeneous support catalyst for easy separation finally it can be utilized in different reactions for the synthesis of organic compounds^10^.

While heterogeneous catalysts sometimes demonstrate extraordinary selectivity, they are often less well-known^11^. Although they are more difficult to design, they are generally more durable and often much easier to separate from products and are recovered for reuse^12^. Therefore, in comparison with homogeneous catalysts, their properties lead to excel, especially in reactions in which the separation of catalyst is regarded as a serious dilemma^13,14^.

Various supports and substrates can be utilized for the synthesis of heterogeneous catalysts for instance, MOF, MCMs, silica, graphene oxide, polymers, etc^15^. but to have high surface area for loading more functional groups, high thermal stability to retain structure in high temperatures and pressure, and high chemical and physical stability are noticeable properties for choosing an appropriate support. Therefore, considering essential properties, a great selection can differentiate the final catalyst from others^16^.

Polymeric graphitic carbon nitride (CN), with appealing properties such as reliable chemical and thermal stability, low density, super hardness, environmentally friendly properties, and wear resistance, is one of the most promising materials for surface modification, photocatalysis, preparation of composites, etc^17–19^. In addition, recently CNs have been employed as suitable support for the preparation of heterogeneous catalysts^20–22^.

**Scheme 1:**
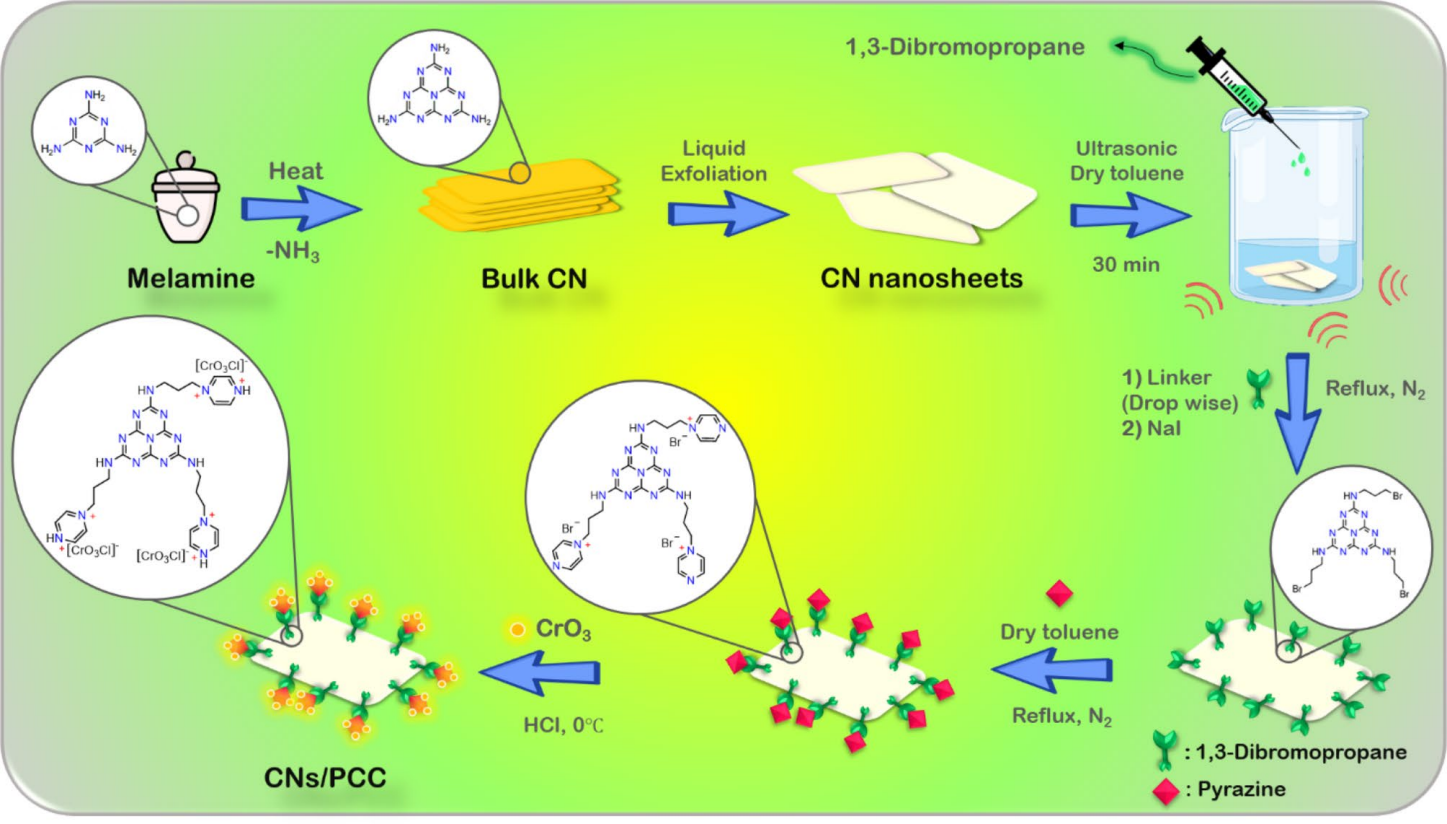
Preparation of the CNs@PCC catalyst.

In this paper, pyrazine was applied instead of pyridine for the synthesis of PCC because of the existence of two active sites of nitrogen to link between support catalyst and chromate. Eventually, the CNs@PCC is synthesized and investigated as an efficient heterogeneous catalyst to generate 1,4-dihydropyridine and 3,4-dihydropyrimidin-2(*1 H*)-one derivatives using benzyl alcohols as primary reactant instead of aldehydes. (Scheme 1)

## Results and discussion

### Characterization of CNs@PCC catalyst

The CN@PCC synthesis method is composed of four stages (Scheme 1**)**. The first stage, polymerization of melamine to bulk g-C_3_N_4_, the second stage, to conversion of bulk into the g-C_3_N_4_^23^, the third stage, the pyrazine was linked to the heterogeneous catalytic substrate for the synthesis of CNs@PCC using 1,3-dibromopropane. Finally, by addition of Cr_2_O_3_. HCl to the previous mixture was prepared by the CNs@PCC catalyst. On the other side, various analyses were performed for the investigation and verification of CNs@PCC heterogeneous catalyst synthesis, such as Fourier Transform Infrared (FT-IR) Spectroscopy, Energy Dispersive Spectrometer (EDS), Field Emission Scanning Electron Microscopy (FE-SEM), and X-ray diffraction analysis (XRD), and Thermal Gravimetric Analysis (TGA).

**Fig. 1:**
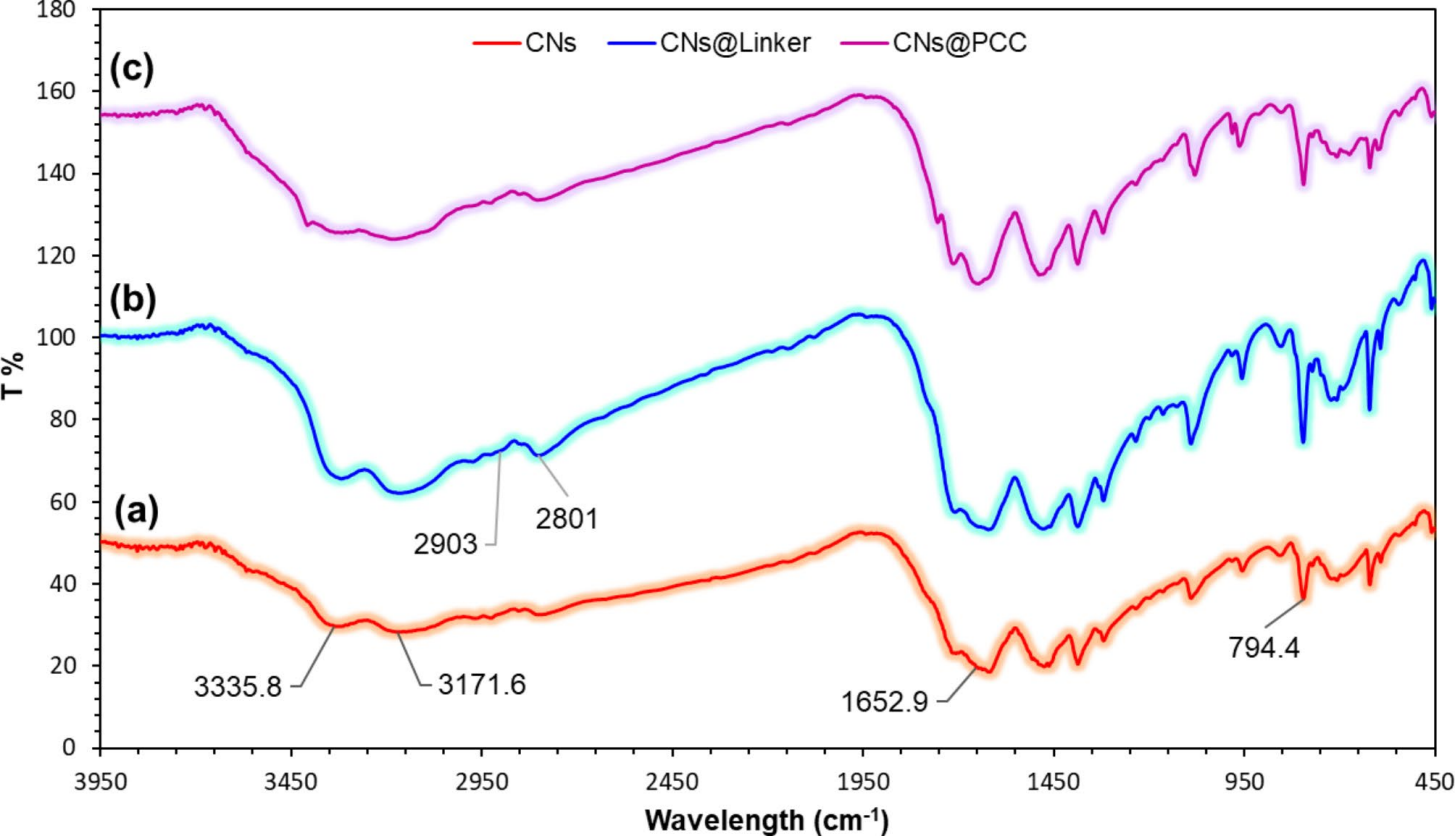
The FT-IR spectra of **(a)** the CNs **(b)** the CNs@linker **(c)** the CNs@PCC.

The identification of different functional groups and bonds in the CNs@PCC was studied using the FT-IR analysis (Fig. 1). As shown in Fig. 1a, the stretching vibration of 794 cm^−1^ is related to the C-N bond of CNs heterogeneous substrate. The appeared peaks in the range of 1300–1500 cm^−1^ are ascribed to the C-N bonds vibration. The strong and broad peak at 1652 cm^−1^can be attributed to the C = N stretching vibration^24,25^. On the other side, the peaks at 3171 and 3335 cm^−1^ were assigned to the N-H stretching mode of primary amine. Moreover, as illustrated in Fig. 1b, the desired adsorption signal at 2801 cm^−1^is related to the C-H aliphatic bond in methylene groups, which can indicate the stabilization of the 1,3-dibromopropane on the CNs surface^26^. The relevant peak to the pyrazine compound was not observed due to the overlap with vibrational peaks of the CNs heterogeneous catalytic substrate (Fig. 1c).

**Fig. 2:**
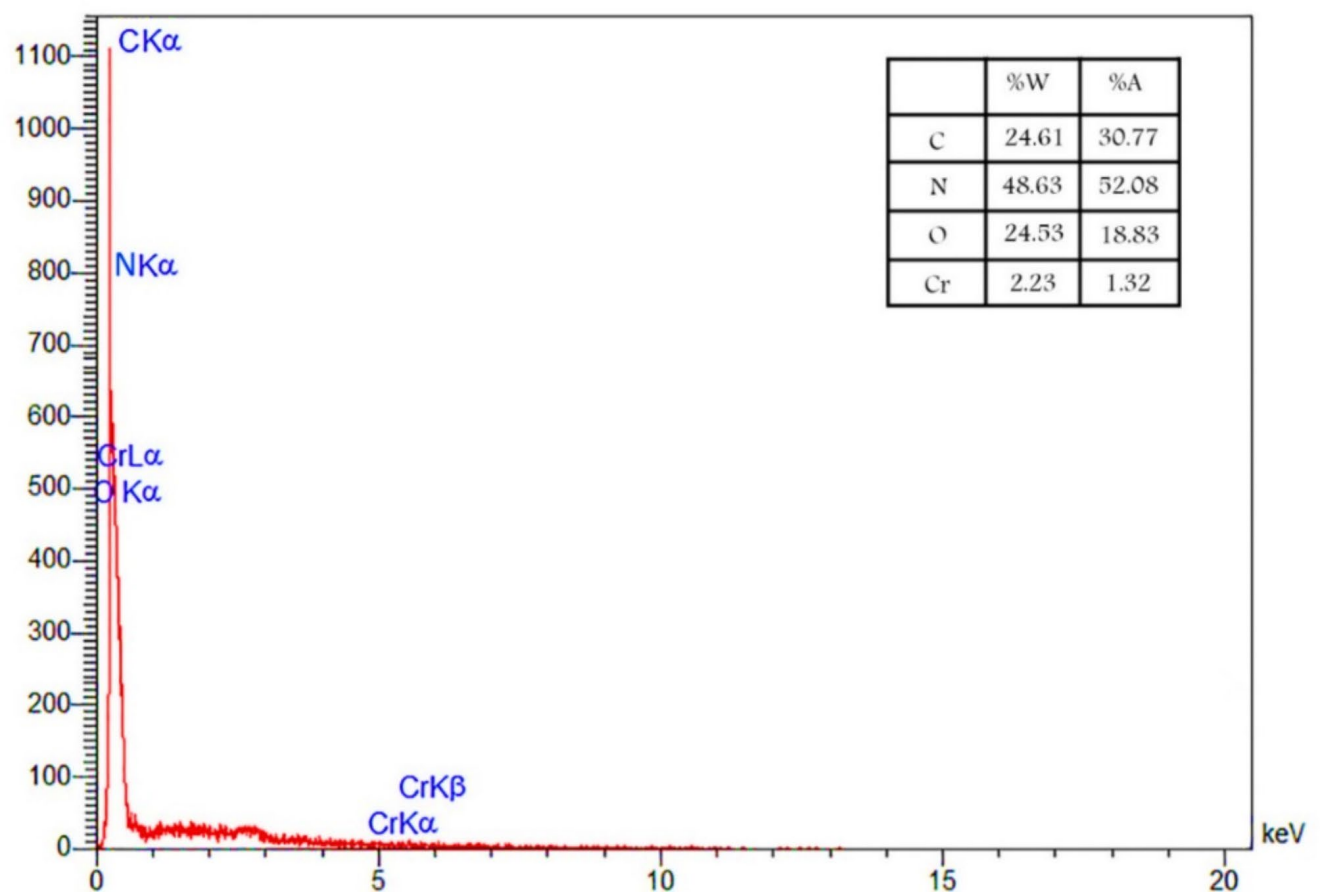
The EDS analysis of the CNs@PCC.

The EDS analysis (Fig. 2) was used to determine the percentage of constituent elements in the catalyst’s structure. The analysis revealed that C and N atoms comprise 24.61% and 48.63% of all elements, respectively. Additionally, the presence of O (24.53%) and Cr (2.23%) elements confirms that the synthesis of CNs@PCC catalyst was successful.

**Fig. 3:**
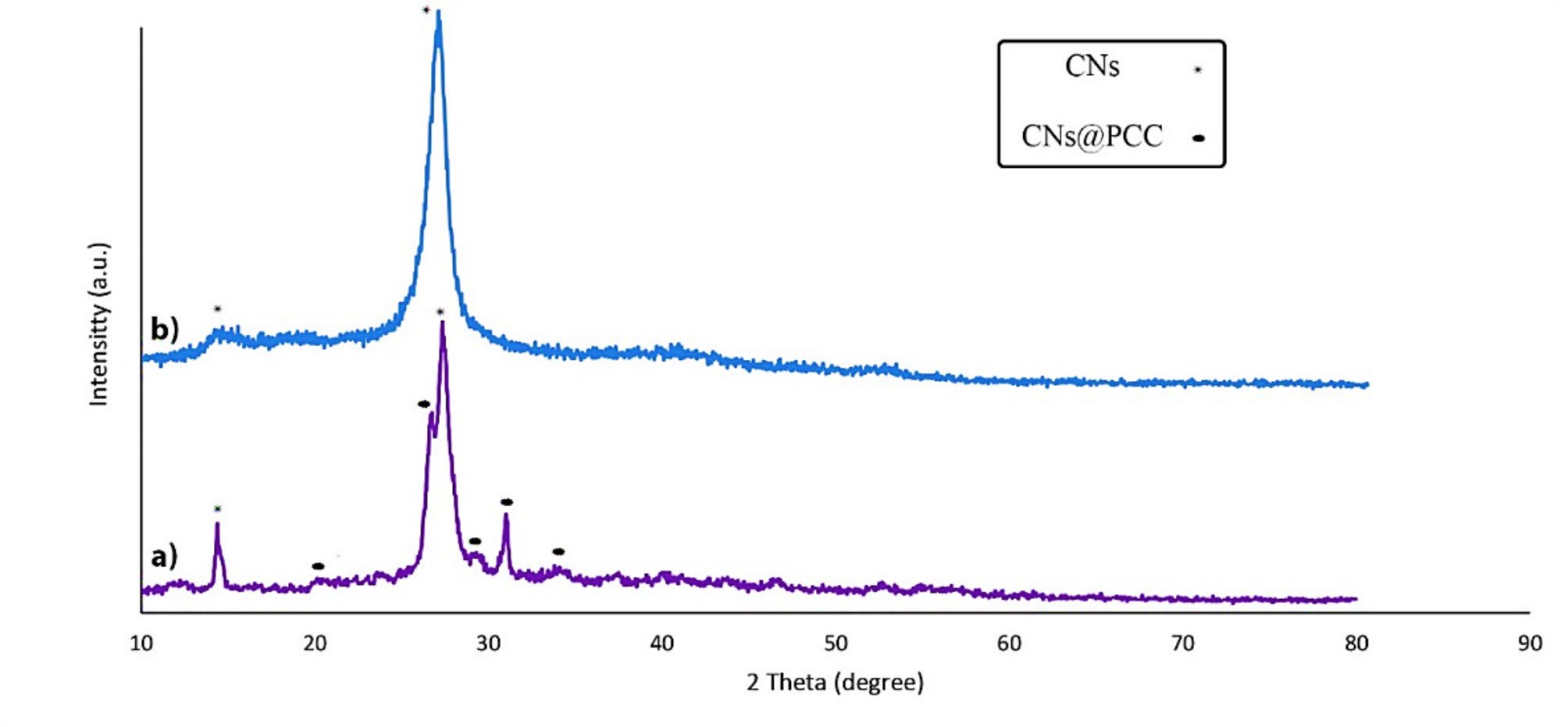
The XRD pattern of the CNs **(a)** and the CNs@PCC **(b)**.

To prove the structure of the heterogeneous catalyst was utilized from the XRD pattern analysis (Fig. 3). The diffraction peaks in the region of 2θ = 27.45° and 2θ = 12.2° are related to CNs (Fig. 3b)^27^, the characteristic diffraction of several peaks at 2θ = 21.39°, 26.13°, 31,32°, 37.63°, and 38.11° corresponded to CrO_3_. Considering the presence of the peaks of CNs in Fig. 3a, the loading of PCC on CNs was verified.

**Fig. 4:**
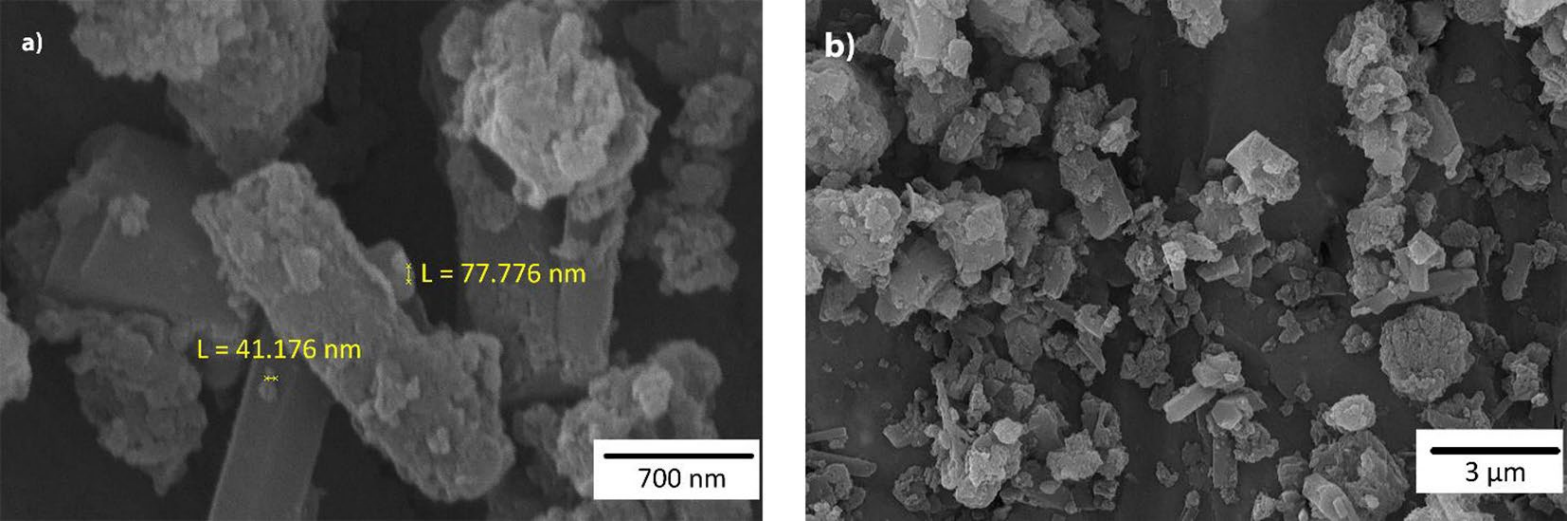
The FE-SEM images of the CNs@PCC (**a** and **b**).

The morphology of the CNs@PCC was investigated by the FE-SEM (Fig. 4a and b). According to the obtained FE-SEM images, the smooth and layered surface of CNs is not completely visible which can be due to the presence of PCC on the surface of CNs^28,29^.

**Fig. 5:**
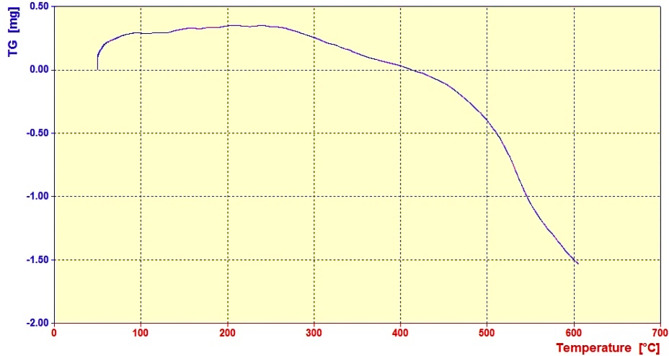
The TGA analysis of the CNs@PCC.

The thermal stability of the CNs@PCC catalyst has been examined by TGA analysis in the range of 50 °C to 800 °C (Fig. 5). As can be monitored in Fig. 5, by increasing the temperature from 200 °C to 400 °C, the weight ratio has decreased, which can be attributed to the separation of pyrazine from the catalyst structure. On the other hand, another weight loss has been reported in the range of 400 °C to 600 °C, which is due to the decomposition of CN heterogeneous support catalyst^30^.

### Model reaction

The performance and efficiency of CNs@PCC catalyst were investigated in benzyl alcohol oxidation for the synthesis of 1,4-dihydropyridine and 3,4-dihydropyrimidin-2-(*1 H*)-one derivatives. In which various parameters such as reaction time, different solvents, temperature, varied oxidants, and amount of catalyst were examined (Table 1). For this target, the synthesis of the product of **5b** for 1,4-dihydropyridine derivatives and the product of **7b** for 3,4-dihydropyrimidin-2-(1* H*)-one derivatives were utilized as model reactions with different conditions.

As shown in Table 1 (**entry 1**), in the non-existence of catalyst after 24 h and at 80 °C, no progress in reaction has been observed. When the CNs@PCC catalyst (10 mg) was added after 4 h the reaction yield was 35% (reaction **a**) and 32% (reaction **b**). Although reaction progress was not noticeable, in comparison with **entry 1** was acceptable. In the next step, various amounts of CNs@PCC catalyst were used to investigate catalyst performance in these reactions under equal conditions (CH_3_CN as solvent, 4 h, 80 °C), the results show 20 mg of catalyst is the optimum amount (**entry 3**,** 4**,** 5**), on the other side, considering final results in **entries 6**,** 7**,** 8**, **9**, **10**, and **11** CH_3_CN solvent can be regarded as the best solvent for these reactions. Then, the influence of other parameters including reaction time, temperature, and oxidant were examined (**entries 12**,** 13**, and **14**).

Eventually, the highest product yield was obtained in the presence of 20.00 mg of CNs@PCC and CH_3_CN as solvent at 80 ℃ under N_2_ atmosphere (**entry 6**). In addition, the TBHP oxidant was added as an oxidant to investigate its effect on reaction yields (**entry 14**), and the PCC reagent was utilized in the non-existence of the catalyst to compare it with catalyst performance (**entry 15**).

As expected, the presence of TBHP as an oxidant was a contributing factor in the development of the reaction, and considering the result of **entry 15**, in comparison with the PCC reagent, the CNs@PCC catalyst displayed a great performance.

Also, as can be seen in Tables 2 and 3, varied derivatives of alcohols were utilized to produce different products (dihydropyridine and dihydropyrimidin-2(1 H)-ones derivatives), each of which had different and significant yields.

**Table 1:** Optimization of various factors for model reactions 1 and 2.

Entry	Catalyst	Catalyst Loading (mg)	Oxidant	Solvent	Time (h)	atmosphere	T(^°^C)	Yield (%)	
								Reaction (1)^(a)^	Reaction (2)^(b)^
1	−	−	−	CH_3_CN	24	N_2_	80	−	−
2	CNs@PCC	10	−	CH_3_CN	4	N_2_	80	35	32
3	CNs@PCC	15	−	CH_3_CN	4	N_2_	80	59	57
4	CNs@PCC	20	−	CH_3_CN	4	N_2_	80	68	70
5	CNs@PCC	30	−	CH_3_CN	4	N_2_	80	69	70
6	CNs@PCC	20	−	CH_3_CN	6	N_2_	80	70	71
7	CNs@PCC	10	−	H_2_O	6	N_2_	80	−	−
8	CNs@PCC	10	−	Ethanol	6	N_2_	80	−	−
9	CNs@PCC	10	−	CH_3_OH	6	N_2_	65	−	−
10	CNs@PCC	10	−	DMF	6	N_2_	90	31	30
11	CNs@PCC	10	−	CHCl_3_	6	N_2_	r.t	68	70
12	CNs@PCC	20	−	CH_3_CN	11	N_2_	r.t	40	43
13	CNs@PCC	20	Et_3_N	CH_3_CN	6	N_2_	80	74	75
14	CNs@PCC	20	TBHP	CH_3_CN	6	N_2_	80	83	87
15	PCC	20	−	CH_3_CN	6	N_2_	80	80	84

^(a)^ Reaction of 4-chlorobenzyl alcohol, ethyl acetoacetate, and NH_4_OAC for the synthesis of 1,4-dihydropyridine. ^(b)^ Reaction of 4-chlorobenzyl alcohol, ethyl acetoacetate, and urea, for synthesis of 3,4-dihydropyrimidin-2(1*H*)-one derivatives.

**Table 2:** Synthesis of 1,4-dihydropyridine derivatives using CNs@PCC nanocatalyst.


Entry	*R*	Product	Time(h)	Mp (°C)	Mp (°C, ref.)	Yield (%)
1	H	5a	4	156–158	155–160	**62**
2	4-Cl	5b	4	144–146	144–146	**69**
3	4-OH	5c	4	229–232	230–232	**67**
4	4-NO_2_	5d	4	130	128–132	**69**
5	4-Me	5e	4	138	136–140	**61**
6	4-OMe	5 g	4	156–160	158–160	**59**

Reaction conditions: benzyl alcohol (1 mmol), ammonium acetate (1 mmol), ethyl acetoacetate (1 mmol), dimedone (1 mmol) CNs@PCC (20 mg) and CH_3_CN (2 mL) under N_2_ atmosphere.

**Table 3:** Synthesis of 3,4-dihydropyrimidinone derivatives using CNs@PCC nanocatalyst.


Entry	R	Product	Time(h)	Mp (°C)	Mp (°C, ref.)	Yield (%)
1	H	7a	4	205-207	203-208	**65**
2	4-Cl	7b	4	212-214	213-215	**70**
3	4-OH	7c	4	209-211	210-213	**61**
4	4-NO_2_	7d	4	204-207	205-207	**67**
5	4-Me	7e	4	208-210	209-210	**58**
6	4-OMe	7f	4	201-203	200-203	**69**

Mechanistic study of the prepared CNs@PCC heterogeneous catalyst for the synthesis of 1,**4-dihyropyridine and 3**,**4-dihydropyrimidin-2-(1*H*)-one derivatives**.

**Scheme 2:**
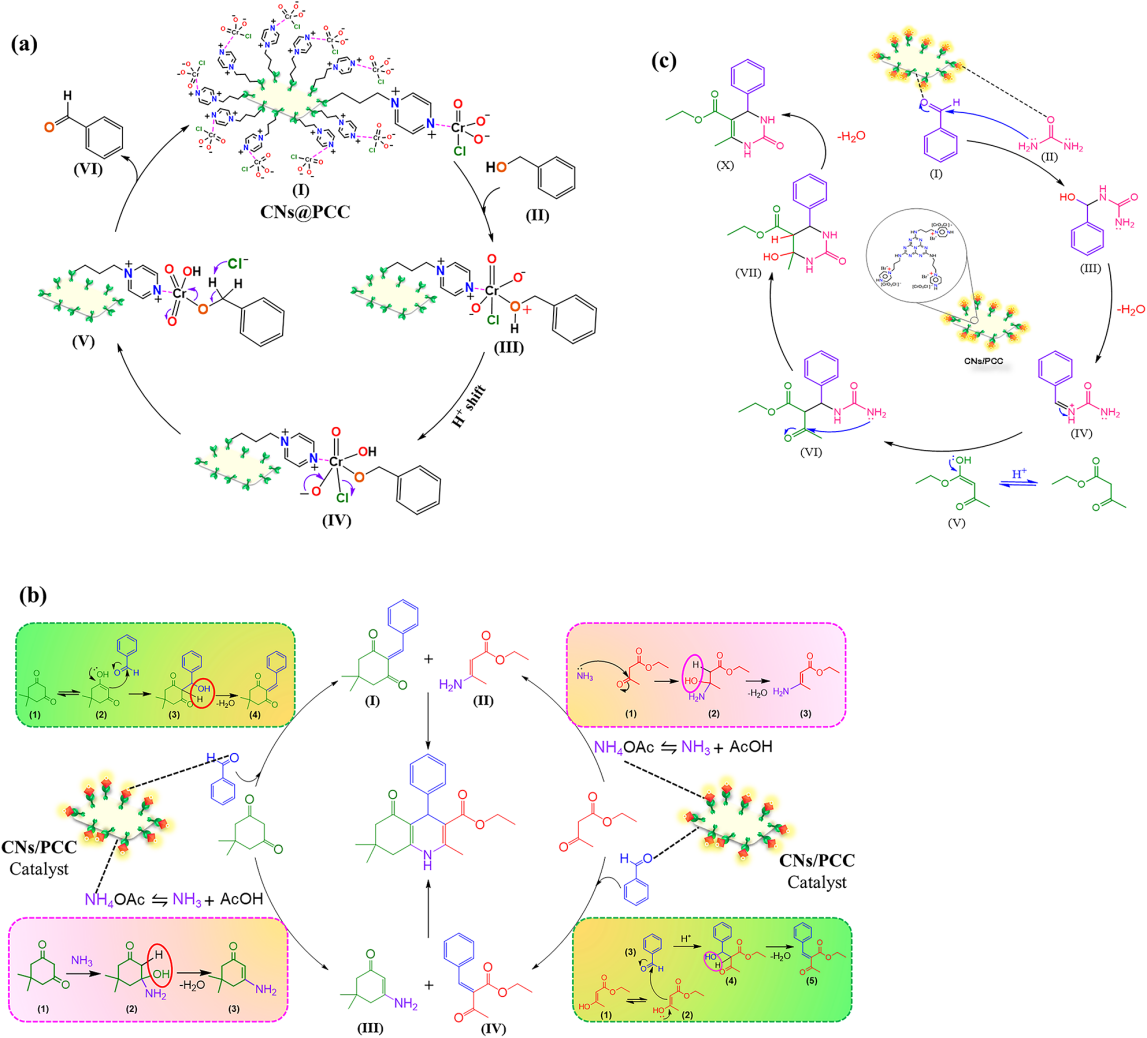
Mechanism for synthesis of different aldehydes from benzyl alcohol derivatives (a), 1,4-dihyropyridine (**b**) and 3,4-dihydropyrimidin-2-(1*H*)-one (**c**) derivatives.

In Scheme 2, the probable and suitable mechanism was suggested for the synthesis of 1,4-dihydropyridine and 3,4-dihydropyrimidin-2-(1*H*)-one derivatives. The first step is similar to both reactions in which benzyl alcohol reacts with a functionalized group of catalyst (Pyrazinium Chlorochromate). In the first step, Oxygen attacks the chromium to form the Cr-O bond. After a proton transfer, a chloride ion is displaced and forms a chromate ester. Following the step, after the elimination of the proton of the carbon, the desired aldehyde is produced, and Cr (VI) is converted to Cr (IV)^31^. In this part, the existence of catalyst lead to oxidation of benzyl alcohol to aldehyde finally reaction progress depends on it, and in absence of catalyst, this process is not feasible and possible **(**Scheme 2a**)**^32^.

As shown in Scheme 2b, for the synthesis of 1,4-dihydropyridine derivatives, In first method, in case reaction between dimedone and aldehyde are formed intermediate **I** and from reaction between ethyl acetoacetate with NH_4_OAC are produced intermediate **II**, but in second method, aldehyde with ethyl acetoacetate produce intermediate **IV** in presence of catalyst (**CNs@PCC**), and dimedone reacts with NH_4_OAC for formation of intermediate **III.** Eventually both methods lead to synthesize desired product (**V**)^33–35^. , the CNs@PCC has been utilized as an activator to reaction progress.

The plausible mechanism for the preparation of 3,4-dihydropyrimidin-2(1*H*)-one derivatives was indicated in Scheme 2c^36,37^. that in which, synthesized aldehyde from oxidation of benzyl alcohol, after activation by catalyst and reaction with urea produce intermediate **III** that subsequent dehydration and reaction with ethyl acetoacetate lead to production of intermediate **VI**, Eventually, the six-member ring is formed by nucleophilic attack and the final product **X** was obtained by the elimination of H_2_O molecule. The presence of catalyst in preparation of 1,4-dihydropyridine and 3,4-dihydropyrimidin-2-(1*H*)-one derivative assist to facilitate this process by declining activation energy of different parts of reaction^38,39^.

## Experimental

### Material

All chemicals were prepared by Sigma-Aldrich and Merck Company. On the other side, the FT-IR spectra were recorded on Tensor 27. The NMR data were acquired on a Varian-Inova 500 MHz. The EDS spectrum was recorded on Numerix DXP–X10P. The XRD pattern was gained by the use of a Dron-8 diffractometer. The FESEM images of CNs@PCC were recorded with TESCAN-MIRA III.

### **Preparation of CNs and modified CNs**

The bulk and CNs were synthesized by the previous reported method at 550 ˚C temperature. The CN (1.0 g) was dispersed completely in dry toluene (20 mL) at 30 min to obtain a homogeneous mixture. Then, NaI (1.0 mmol) was added to the solution, and after the addition of 1,3-dibromopropane (2.0 mL, dropwise), the reaction mixture was refluxed under N_2_ atmosphere for 24 h. Finally, the obtained mixture (Product **A**) was washed several times with ethyl acetate solvent and dried at 60 ˚C. After that, product **A** (10 mg) was dispersed in dry toluene (20.0 mL) then the pyrazine (150 µL) was added and stirred under N_2_ atmosphere at 100 ˚C for 24 h. The resulting product was filtered and washed with ethyl acetate, then dried at room temperature (Product **B**).

### **Preparation of CNs@PCC**

For the synthesis of CNs@PCC catalyst. Initially, HCl (5.0 mL, 12 M) was added to the Chromium trioxide (CrO_3_) (1.0 g) and was stirred at room temperature for 15 min (the mixture discolored to red). Then, the obtained mixture was cooled to 0 °C, and product **B** was added to it and has been stirred at room temperature for 24 h (the mixture discolored from red to green). Finally, the synthesized catalyst (product **C**) was washed with DI water and dried at 70 °C.

### **General procedure for the synthesis of 1**,**4-dihydropyridine derivatives via oxidation of benzyl alcohols by CNs@PCC**

A mixture of alcohol (1.0 mmol), ethyl acetoacetate (1.0 mmol), NH_4_OAc (1.0 mmol), dimedone (1.0 mmol), CNs@PCC (20 mg), and 2 mL of CH_3_CN as a reaction solvent was stirred under N_2_ atmosphere at 80 °C for 4 h in an oil bath. The progress of the reaction was monitored using Thin Layer Chromatography (TLC). After the reaction was complete, the heterogeneous catalyst was separated by filtration and washed with ethyl acetate. The resulting solid was dissolved in ethyl acetate (10 mL) and added to H_2_O (20 mL) and NaCl (50 mg). The organic phase was then separated from the aqueous phase and recrystallized by EtOH/H_2_O. The CNs@PCC heterogeneous catalyst was dried and reused for the next run.

### **General procedure for the synthesis of 3**,**4-dihydropyrimidin-2-(1 H)-one derivatives via oxidation of benzyl alcohols by CNs@PCC**

The mixture of benzyl alcohol (1.0 mmol), ethyl acetate (1.0 mmol), urea (1.0 mmol), CNs@PCC (20 mg), and 2 mL of CH_3_CN solvent was stirred under N_2_ atmosphere at 80 °C for 4 h. The next steps were the similar synthesis of 1, 4-dihydropyrimidine derivatives. After completion of the reaction, the heterogeneous catalyst was separated and washed with ethyl acetate. After evaporation of CH_3_CN, the resulting solid was dissolved in ethyl acetate (10 mL) and added to H_2_O (20 mL) and NaCl (50 mg). Finally, the organic phase was separated from the aqueous phase and recrystallized by EtOH/H_2_O, and the CNs@PCC heterogeneous catalyst was dried and reused for the next run.

## Conclusion

In conclusion, while there are different methods to convert alcohols to aldehydes, the selection of the best approach can be a serious challenge, therefore, in this work, CNs@PCC was designed for this purpose and was employed as a heterogeneous catalyst in the oxidation of benzyl alcohol derivatives to corresponding aldehydes for the synthesis of 1,4-dihydropyridine and 3,4-dihydropyrimidin-2-(1*H*)-one derivatives. On the other side, the successful synthesis of CNs@PCC catalyst was confirmed by various analyses such as FT-IR, EDS, XRD, TGA, and FE-SEM. Finally, the following points can be mentioned from the important achievements of this work;

1. Novel synthesis of the P.C.C on a solid support catalyst (CNs) in order to easy separation.
2. The conversion of benzyl alcohols to aldehydes and synthesis of 4-dihydropyridine and 3,4-dihydropyrimidin-2-(1*H*) one derivatives simultaneously.
3. The usage of benzyl alcohols instead of corresponding aldehydes as reactants in production of 3,4-dihydropyrimidin-2-(1*H*) one derivatives.

## Electronic supplementary material

Below is the link to the electronic supplementary material.

Supplementary Material 1

## Data Availability

All data generated or analysed during this study are included in this published article [and its supplementary information files].
