# Supplementary material for: Efficient in-situ synthesis of heterocyclic derivatives from benzyl alcohols using pyrazinium chlorochromate-functionalized carbonitride as a novel catalyst
Source: Sci Rep. 2024 Oct 14;14:23987. doi: 10.1038/s41598-024-75036-6 (PMC11473725; doi:10.1038/s41598-024-75036-6)
Supplement: Supplementary file 1 — Supplementary Material 1 [file 41598_2024_75036_MOESM1_ESM.docx]

**Efficient *in-situ* synthesis of heterocyclic derivatives from benzyl alcohols using pyrazinium chlorochromate-functionalized carbonitride as a novel catalyst**

*Catalysts and Organic Synthesis Research Laboratory, Department of Chemistry, Iran University of Science and Technology, Tehran16846-13114, Iran*

**Corresponding author E-mail:* [*ghafuri@iust.ac.ir*](mailto:ghafuri@iust.ac.ir)*; Fax: +98-21-77491204; Tel: +98-21-77240516-7*

**Preparation of CNs and CNs@PCC**

The bulk and CNs were synthesized by the previous reported method at 550 ˚C temperature. The CN (1.0 g) was dispersed completely in dry toluene (20 mL) at 30 min to obtain a homogeneous mixture. Then, NaI (1.0 mmol) was added to the solution, and after the addition of 1,3-dibromopropane (2.0 mL, dropwise), the reaction mixture was refluxed under N_2_ atmosphere for 24 h. Finally, the obtained mixture (Product **A)** was washed several times with ethyl acetate solvent and dried at 60 ˚C. After that, product **A** (10 mg) was dispersed in dry toluene (20.0 mL) then the pyrazine (150 μL) was added and stirred under N_2_ atmosphere at 100 ˚C for 24 h. The resulting product was filtered and washed with ethyl acetate, then dried at room temperature (Product **B**). For the synthesis of CNs@PCC catalyst. Initially, HCl (5.0 mL, 12 M) was added to the Chromium trioxide (CrO_3_) (1.0 gr) and was stirred at room temperature for 15 min (the mixture discolored to red). Then, the obtained mixture was cooled to 0 °C, and product **B** was added to it and has been stirred at room temperature for 24 h (the mixture discolored from red to green). Finally, the synthesized catalyst (product **C**) was washed with DI water and dried at 70 °C (**Scheme S1**).


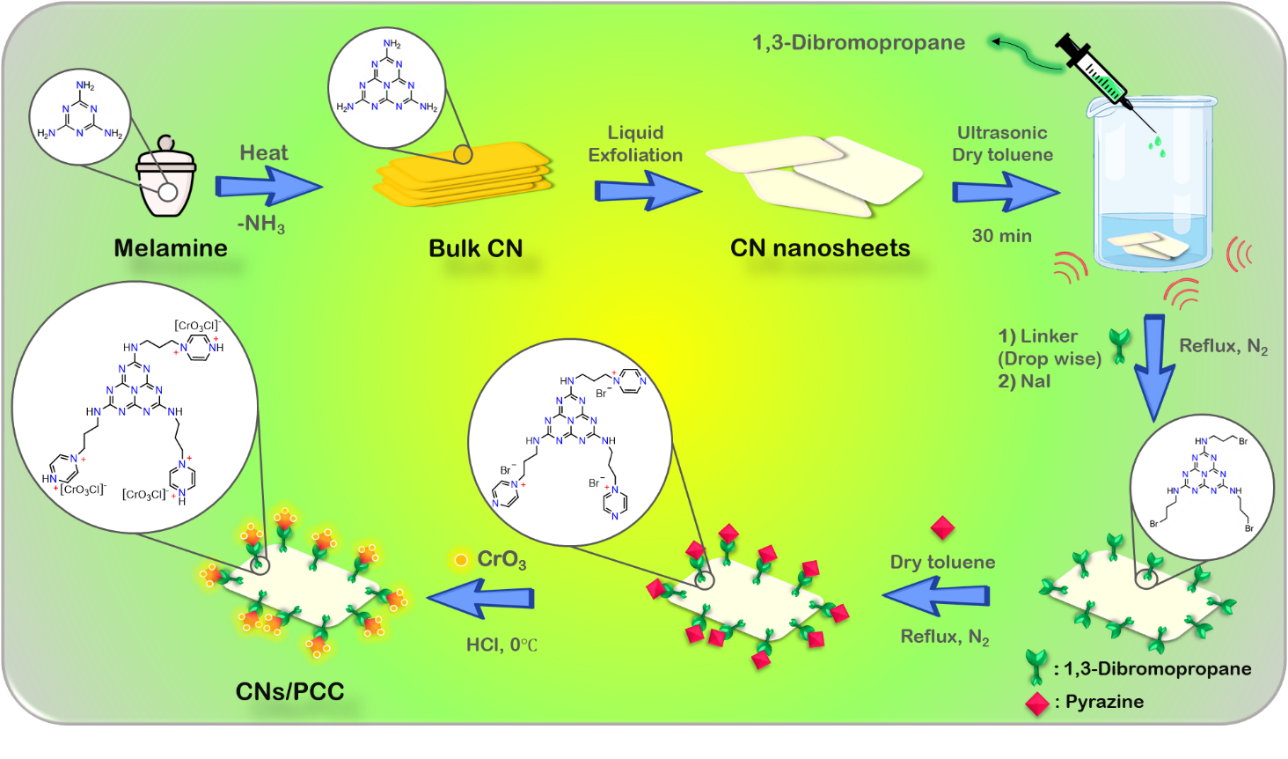


**Scheme S1**. Preparation of the CNs@PCC catalyst.

**Characterization of the CNs@PCC catalyst.**


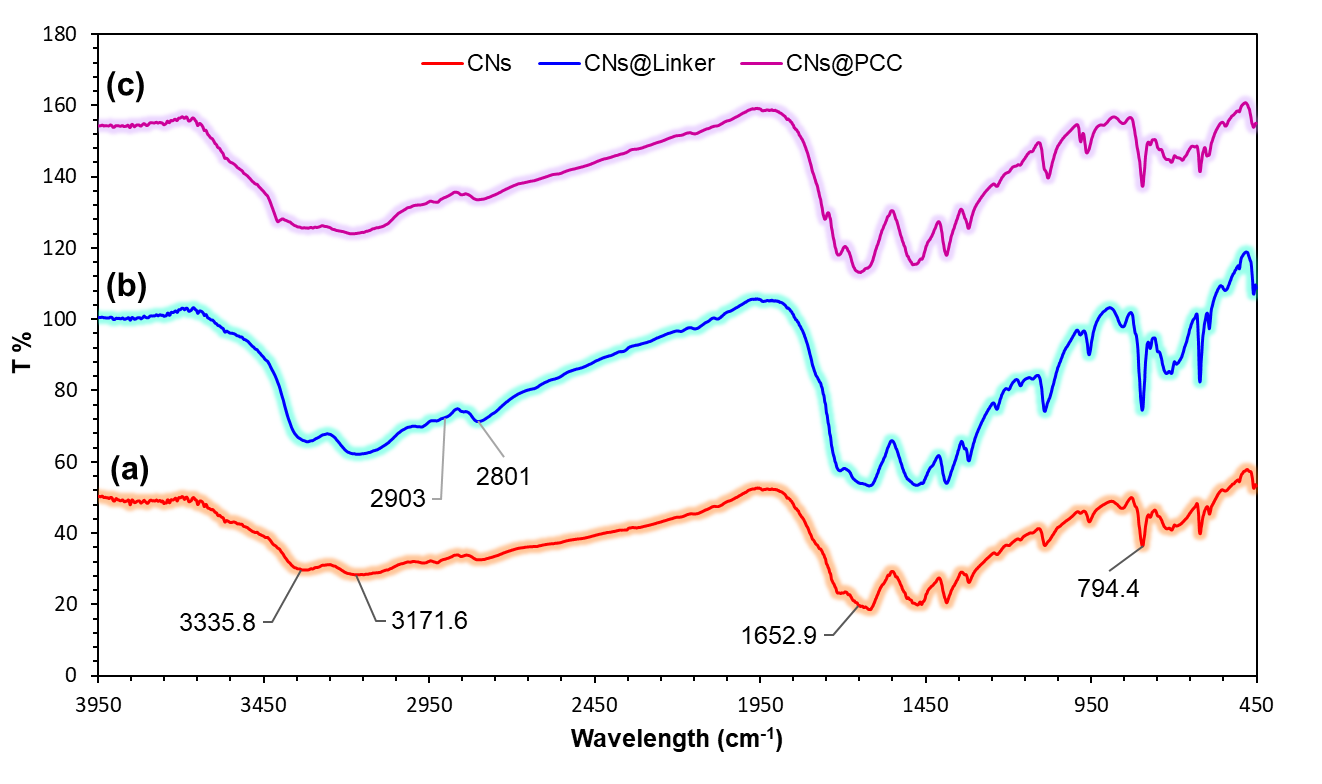


**Fig S1**. The FT-IR spectra of **(a)** the CNs **(b)** the CNs@linker **(c)** the CNs@PCC.


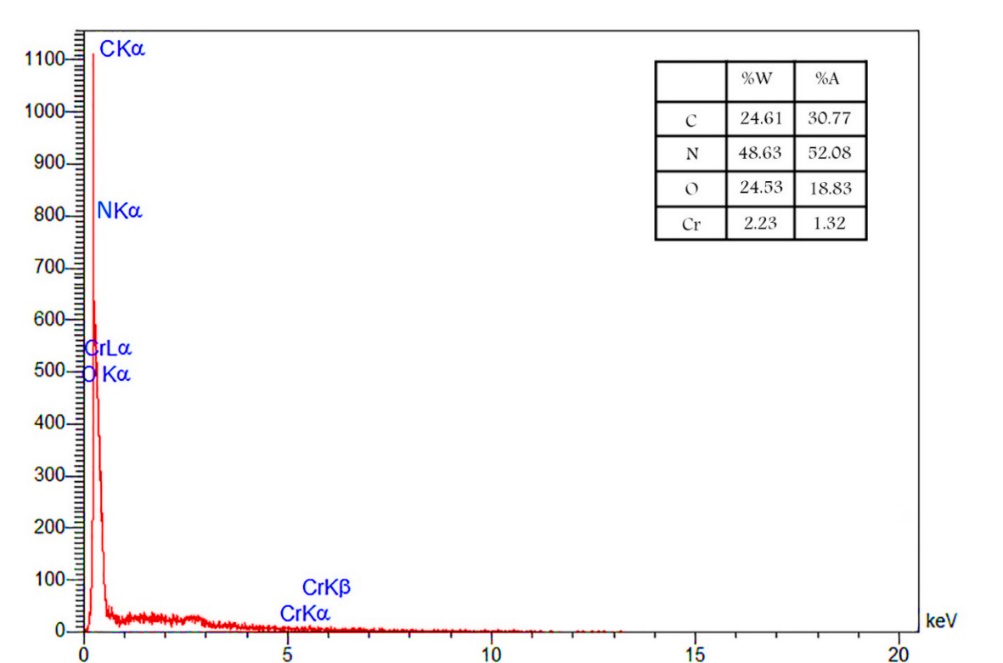


**Fig S2**. The EDS analysis of the CNs@PCC.


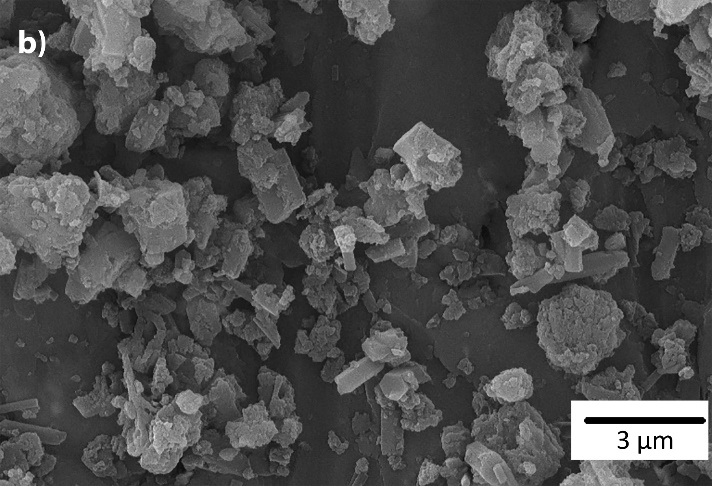

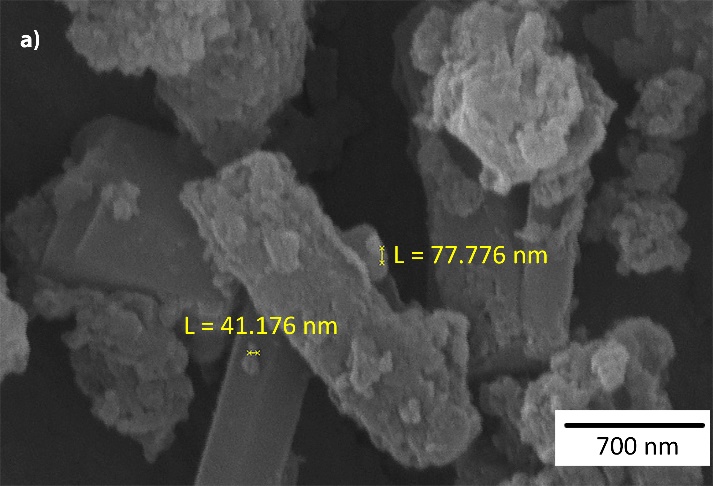


**Fig S3**. The FE-SEM images of the CNs@PCC (**a** and **b**).


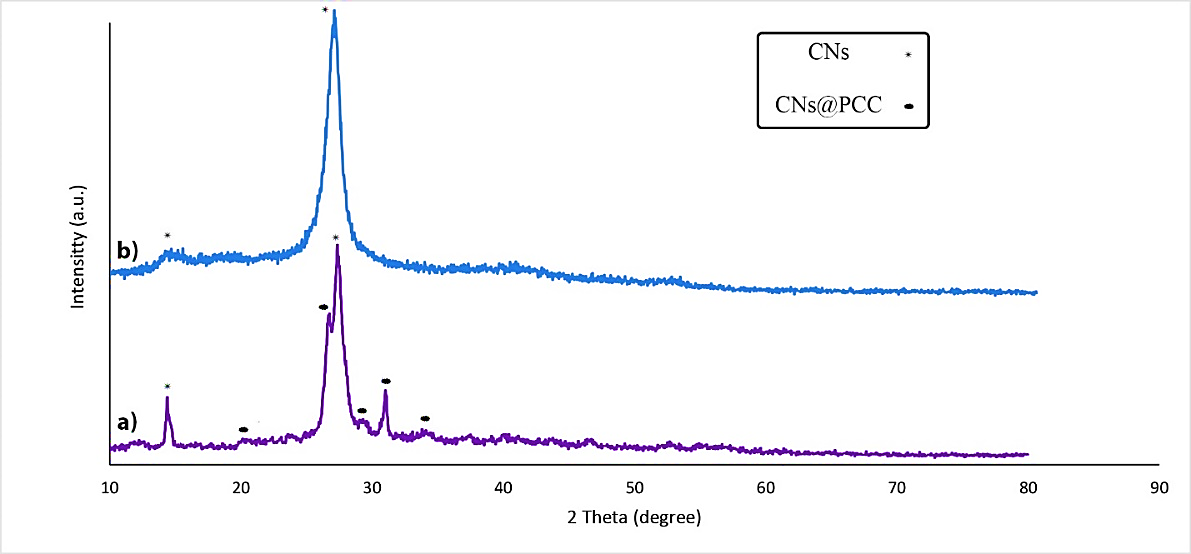


**Fig S4.** The XRD pattern of the CNs **(a)** and the CNs@PCC **(b)**.

**
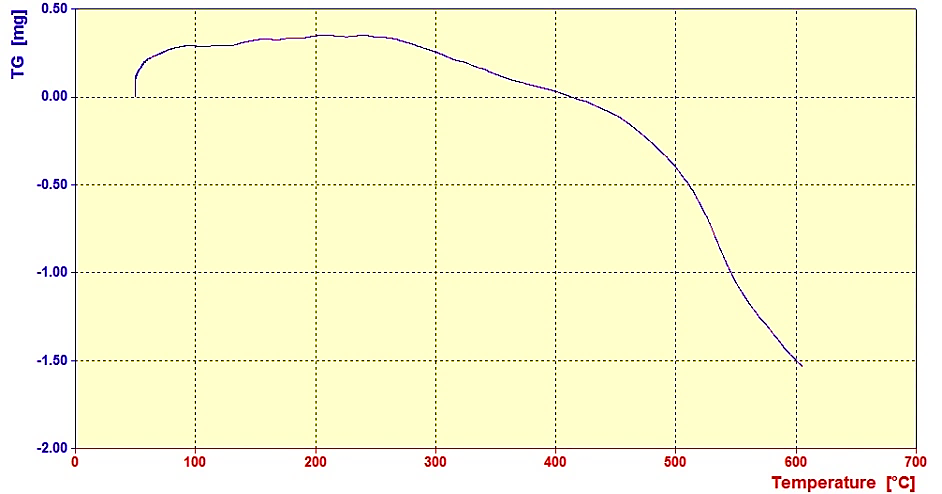
**

**Fig S5**. The TGA analysis of the CNs@PCC.

**General procedure for the synthesis of 1,4-dihydropyridine derivatives via oxidation of benzyl alcohols by CNs@PCC.**

A mixture of alcohol (1.0 mmol), ethyl acetoacetate (1.0 mmol), NH_4_OAc (1.0 mmol), dimedone ( 1.0 mmol), CNs@PCC (20 mg), and 2 mL of CH_3_CN as a reaction solvent was stirred under N_2_ atmosphere at 80 °C for 4h in an oil bath. The progress of the reaction was monitored using Thin Layer Chromatography (TLC). After the reaction was complete, the heterogeneous catalyst was separated by filtration and washed with ethyl acetate. The resulting solid was dissolved in ethyl acetate (10 mL) and added to H_2_O (20 mL) and NaCl (50 mg). The organic phase was then separated from the aqueous phase and recrystallized by EtOH/H_2_O. The CNs@PCC heterogeneous catalyst was dried and reused for the next run.

**General procedure for the synthesis of 3,4-dihydropyrimidin-2-(1*H*)-ones derivatives via oxidation of benzyl alcohols by CNs@PCC**.

The mixture of benzyl alcohol (1.0 mmol), ethyl acetate (1.0 mmol), urea (1.0 mmol), CNs@PCC (20 mg), and 2 mL of CH_3_CN solvent was stirred under N_2_ atmosphere at 80 °C for 4h. The next steps were the similar synthesis of 1, 4-dihydropyrimidine derivatives. After completion of the reaction, the heterogeneous catalyst was separated and washed with ethyl acetate. After evaporation of CH_3_CN, the resulting solid was dissolved in ethyl acetate (10 mL) and added to H_2_O (20 mL) and NaCl (50 mg). Finally, the organic phase was separated from the aqueous phase and recrystallized by EtOH/H_2_O, and the CNs@PCC heterogeneous catalyst was dried and reused for the next run.

**Reusability of the CNs@PCC heterogeneous catalyst**

The recovery and reusability of catalysts is a beneficial principle in green chemistry. Therefore, the recyclability of CNs@PCC heterogeneous catalyst was studied in the synthesis of 1,4-dihydropyridine (**5b**) and 3,4-dihydropyrimidin-2-(1H)-one (**7b**) products. For this purpose, after completion of the reaction, the catalyst was separated and washed with H_2_O and EtOH, then dried and reused four times. Although in each run, some amount of the reaction yield has decreased especially in the last run, but results were acceptable (**Fig S6**). As indicated in **Fig S7**, the FT-IR spectrum of recycled CNs@PCC heterogeneous catalyst proves that the initial structure of the catalyst has been preserved after four times use.

**Fig S6.** Examination of CNs@PCC reusability in synthesis of 1,4-dihydropyridine (**5b**) and 3,4-dihydropyrimidin-2-(1*H*)-one (**7b**).

**Fig S7.** The FT-IR spectrum of CNs@PCC catalyst after the five-times recycling.

**Selected Spectral Data:**

**1. Ethyl 2, 7, 7-trimethyl-5-oxo-4-(4-hydroxylphenyl)-1,4,5,6,7,8-hexahydroquinoline-3-carboxylate (5c)**

FTIR (KBr, cm-1): 3270, 3194, 3071, 2957, 1678, 1645, 1481, 1377, 1214 cm-1. 1H NMR (500 MHz,

DMSO): δ H (ppm)= 0.85(s, 3H, CH3), 1.0(s, 3H, CH3), 1.13(t, 3H, CH3), 1.9-2.41(m,4H, 2CH2), 2.25(s, 3H, CH3), 3.95-3.99(q, 2H, OCH2), 4.73(s, 1H, Ar-CH), 6.54(d, 2H, Ar-H), 6.93(d, 2H, Ar-H), 8.95(s, 1H, NH), 9.01(s,1H, OH).

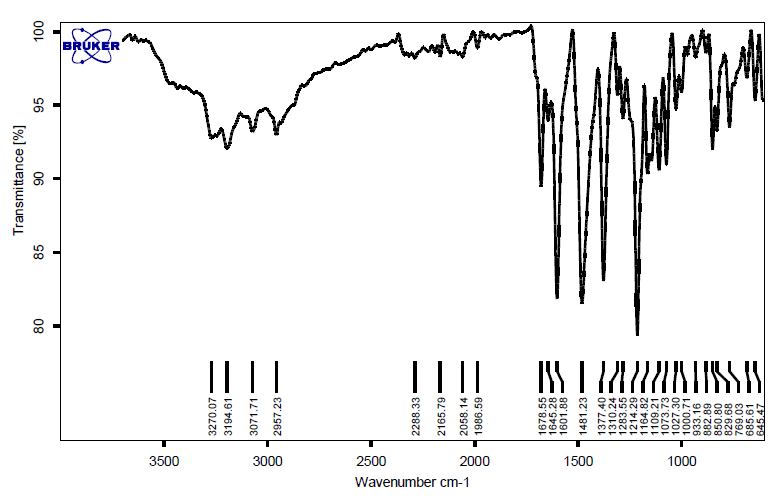


**Fig S8**. FT-IR spectrum of the Ethyl 2, 7, 7-trimethyl-5-oxo-4-(4-hydroxylphenyl)-1,4,5,6,7,8-hexahydroquinoline-3-carboxylate (5c)


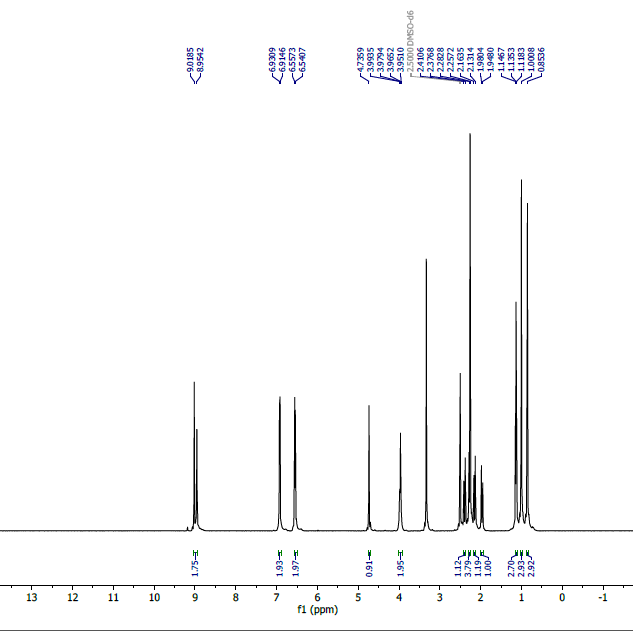


**Fig S9.** ^1^HNMR spectrum of the Ethyl 2, 7, 7-trimethyl-5-oxo-4-(4-hydroxylphenyl)-1,4,5,6,7,8-hexahydroquinoline-3-carboxylate (**5c**)

**2. Ethyl 1,4,7,8-tetrahydro-2,7,7-trimethyl-4-(4-nitrophenyl)-5(6H)-oxoquinoline-3-carboxylate (5d)**

FTIR (KBr, cm-1): 3276, 3210, 3076, 2969, 2902, 1703, 1641, 1530, 1379 cm-1. 1H NMR (500 MHz, DMSO): δH (ppm)= 0.83(s, 3H, CH3), 1.01(s, 3H, CH3), 1.11(t, 3H, CH3), 1.96-2.46(m,4H, 2CH2), 2.31(s, 3H, CH3), 3.93-4.0(m, 2H, OCH2), 4.97(s, 1H, Ar-CH), 7.5-7.61 (m, 4H, Ar-H), 7.97(s, 1H, NH), 9.23(s,1H, OH).

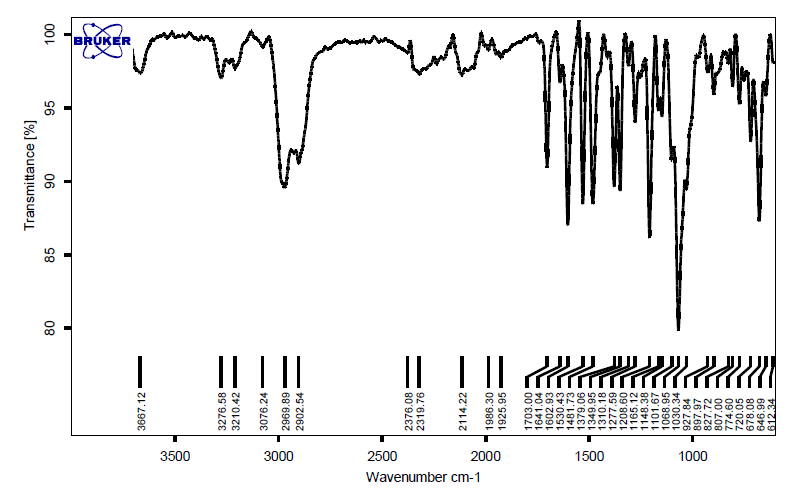


**Fig S10.** FT-IR spectrum of the Ethyl 1,4,7,8-tetrahydro-2,7,7-trimethyl-4-(4-nitrophenyl)-5(6H)-oxoquinoline-3-carboxylate (5d).


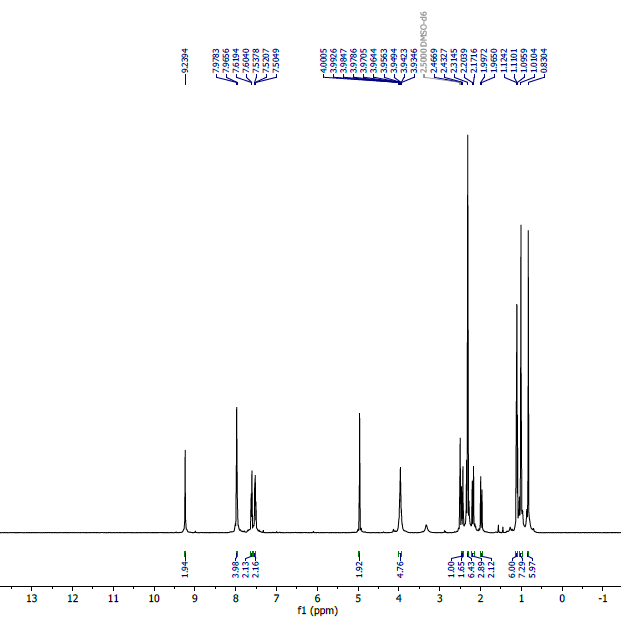


**Fig S11.** ^1^HNMR spectrum of the Ethyl 1,4,7,8-tetrahydro-2,7,7-trimethyl-4-(4-nitrophenyl)-5(6H)-oxoquinoline-3-carboxylate (**5d**)

**3. 3, 4-dihydro-5-ethoxy carbonyl-4-(4-phenyl)-6-methyl-pyrydine-2-(1H) one (7a)**

MP: 205-207. FTIR (KBr.cm^-1^): 3200, 3100, 2975, 1650, 1460, 1290, 1215, 7601216 cm^-1^.^1^H NMR (500 MHz, DMSO): ᵟ H (ppm) = 1.09 (t, CH_3_), 2.24 (s, allyl), 3.9 (q, CH_2_), 5.1 (d, Ar-CH), 7.24-7.31 (m, Ar-H), 7.72-9.1 (s, N-H).

**
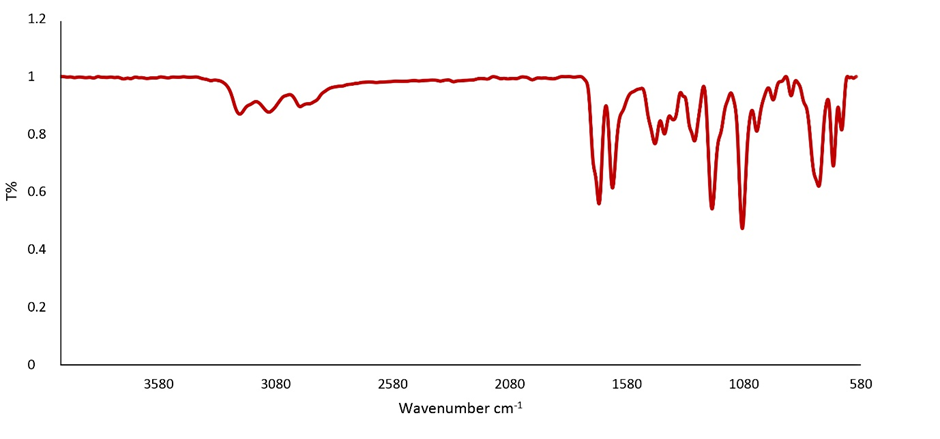
**

**Fig S12**. The FT-IR spectrum of the 3, 4dihydro-5-ethoxy carbonyl-4-(4-phenyl)-6-methyl-pyrydine-2-(1H) one (7a).


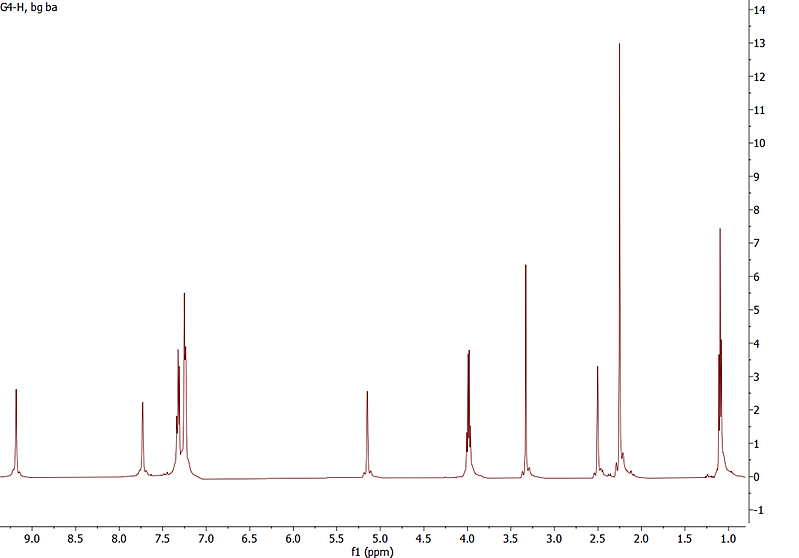


**Fig S13**. The ^1^HNMR spectrum of of the 3, 4dihydro-5-ethoxy carbonyl-4-(4-phenyl)-6-methyl-pyrydine-2-(1H) one (7a)
